# Ultrasonic biomechanics method for vortex and wall motion of left ventricle: a phantom and in vivo study

**DOI:** 10.1186/s12872-021-02317-7

**Published:** 2021-10-24

**Authors:** Aohua Zhang, Min Pan, Long Meng, Fengshu Zhang, Wei Zhou, Yaonan Zhang, Rongqin Zheng, Lili Niu, Yanling Zhang

**Affiliations:** 1 Department of Ultrasound, Third Affiliated Hospital, Sun Yat-Sen University, 600 Tianhe Road, Guangzhou, 510630 Tianhe District China; 2 Department of Ultrasound, Shenzhen Hospital (Futian) of Guangzhou University of Chinese Medicine, Shenzhen, China; 3 Paul C. Lauterbur Research Center for Biomedical Imaging, Institute of Biomedical and Health Engineering, Shenzhen Institutes of Advanced Technology, Chinese Academy of Sciences, Shenzhen, China; 4 Sino-Dutch Biomedical and Information Engineering School, Northeastern University, Shenyang, China

**Keywords:** Ultrasonic imaging, Wall motion, Vortex, Strain, Left ventricular dysfunction

## Abstract

**Background:**

The non-invasive quantitative evaluation of left ventricle (LV) function plays a critical role in clinical cardiology. This study proposes a novel ultrasonic biomechanics method by integrating both LV vortex and wall motion to fully assess and understand the LV structure and function. The purpose of this study was to validate the ultrasonic biomechanics method as a quantifiable approach to evaluate LV function.

**Methods:**

Firstly, B-mode ultrasound images were acquired and processed, which were utilized to implement parameters for quantifying the LV vortex and wall motion respectively. Next, the parameters were compared in polyvinyl alcohol cryogen (PVA) phantoms with different degree of stiffness corresponding to different freezing and thawing cycles in vitro. Finally, the parameters were computed in vivo during one cardiac cycle to assess the LV function in normal and abnormal subjects in vivo.

**Results:**

In vitro study, the velocity field of PVA phantom differed with stiffness (varied elasticity modulus). The peak of strain for wall motion decreases with the increase of elasticity modulus, and periodically changed values. Statistical analysis for parameters of vortex dynamics (energy dissipation index, DI; kinetic energy fluctuations, KEF; relative strength, RS; and vorticity, W) based on different elasticity (E) of phantom depicted the good viability of this algorithm. In vivo study, the results confirmed that subjects with LV dysfunction had lower vorticity and strain (S) compared to the normal group.

**Conclusion:**

Ultrasonic biomechanics method can obtain the vortex and wall motion of left ventricle. The method may have potential clinical value in evaluation of LV dysfunction.

## Background

There is an increasing risk of sudden death from cardiac diseases among survivors of acute myocardial infarction with reduced left ventricular (LV) systolic function [1]. Quantification of LV function is important for prognosis evaluation and clinical description of clinical characteristics of patients with multiple forms of cardiac disease [2]. The non-invasive cardiovascular imaging technologies are becoming the focus of interest in the field. It is advantageous to evaluate LV function accurately in order to understand the mechanical abnormity that may lead to LV dysfunction.

During early diastolic filling, the transmitral blood flow is directed towards the left ventricle, which leads to formation of a vortex. Vorticity (W) is an essential parameter of the fluid structure, defined as the curl of velocity field, which can be calculated by the gradient component of velocity in different directions [3]. The presence of vorticities have been recognized by the flow-based visualization techniques both in vitro models [3] and in vivo experiments [3–5] of human left ventricle, or by color Doppler mapping [6] and magnetic resonance velocity mapping [7]. In a healthy left ventricle, the intraventricular flow pattern has a natural structure to minimize the energy dissipation, so that the occurrence of abnormal vorticities may alter the momentum transfer in blood flow and increase energy consumption during ejection [8, 9]. In other words, any disorder in such a natural arrangement can cause LV dysfunction based on underlying fluid dynamics. The vortical flow is thus potentially a novel indicator of LV dysfunction that has not been adopted in previous studies. Much research work has already been conducted to evaluate and predict the overall cardiac health status based on quantitative parameters describing LV function. These parameters are vorticity (W), wall shear stress (WSS), relative strength (RS), energy dissipation index (DI), kinetic energy fluctuation (KEF), and vortex fluctuation. They are considered as critical indicators for detecting and monitoring abnormities of vortex dynamics with high implications on patient’s LV dysfunction [3].

However, vortex dynamics seeded by particles [10] primarily focused on the behavior of LV blood flow. In fact, it is indicated that the changes in either LV morphology or LV contractile ability may alter intraventricular fluid flow pattern and form vortex ring [11]. Previously, numerous attempt was proposed to define LV myocardial function by quantitatively assessing regional myocardial deformation in normal and abnormal segments [12], allowing for noninvasive estimation of the indicators for regional LV wall motion [13].

Simultaneously, strain (S) and strain rate (SR), directly reflect regional myocardial deformation with novel indexes that are building blocks in assessing LV wall performance [14–16]. Here, S is a dimensionless quantity and describes the deformation produced by stress; it represents the percentage change in size compared to original length. The SR is also equivalent to the rate of deformation that can be calculated by spatial velocity gradient. Several investigations have demonstrated the feasibility of S and SR imaging to quantitatively characterize myocardial function [14, 17]. There are several studies which discuss about the measurement of S and SR by tissue Doppler imaging (DTI) techniques, but suffering from the limitation of angle dependence either in vitro or in vivo [18, 19]. The recent research works have proposed novel frame-by-frame techniques, to track speckles in two-dimensional echocardiography gray-scale images, for overcoming the angel dependence of tissue Doppler imaging [20, 21]. Speckle tracking is a method that is suitable for measuring the myocardial motion with speckle tracking patterns in ultrasound B-mode acquisitions [22].

From all the aforementioned works, it is observed that the S and SR are playing an important role in assessing and evaluating the myocardial function. However, few studies attempt to take the vortex dynamics and the myocardial motion of left ventricle into account for integrated assessment of LV function. This study proposes a novel ultrasonic biomechanics method by integrating both LV functions (the vortex dynamics and the myocardial motion of left ventricle) to fully assess and understand the mechanisms in LV structure and function. Compared with conventional processes, We improved the resolution, accuracy, and operation rate in our method by employing numerical techniques including: cross-correlation calculation, Gaussian multimodal fitting, pseudo-vectoring and zero-vector processing, Taylor expansion and multiple iterations in window deformation, and the simplification of the diagnostic window matrix. This method serves as a useful application to analyze the vortex and regional wall motion of LV. It will guide individuals to obtain two-dimensional LV indices and to assess LV function precisely. The purpose of the present study is to validate the ultrasonic biomechanics method as a quantifiable approach to evaluate LV function.

## Methods

### Ultrasonic biomechanics method

In this study, we propose a novel method for analyzing and evaluating the ventricular vortex and wall motion from B-mode echocardiograms. B-mode ultrasound images were obtained and then analyzed with Matlab software, using the cross-correlation calculation that has been described and validated by Niu et al. [24] in our team. The pairs of successive digital images were obtained to compute the magnitude and direction of flow. This technique focused on the relevant regions of the ventricle, so the velocity vector was extracted inside a user-defined region of interest (ROI) in sequential frames, with boundary conditions considered. The entire cavity of LV was selected as the ROI for analyzing the vortical flow dynamics, and different segments of the wall were selected as ROIs for corresponding wall motion study. Every ROI was divided into a regular grid, which comprises interrogation windows. We were evaluating the displacement of ROI in successive frames with maximum cross correlation coefficient between the original and substituted ROIs. An interrogation window of 52 × 52 pixels with 50% overlap was used for the calculation.

**Fig. 1:**
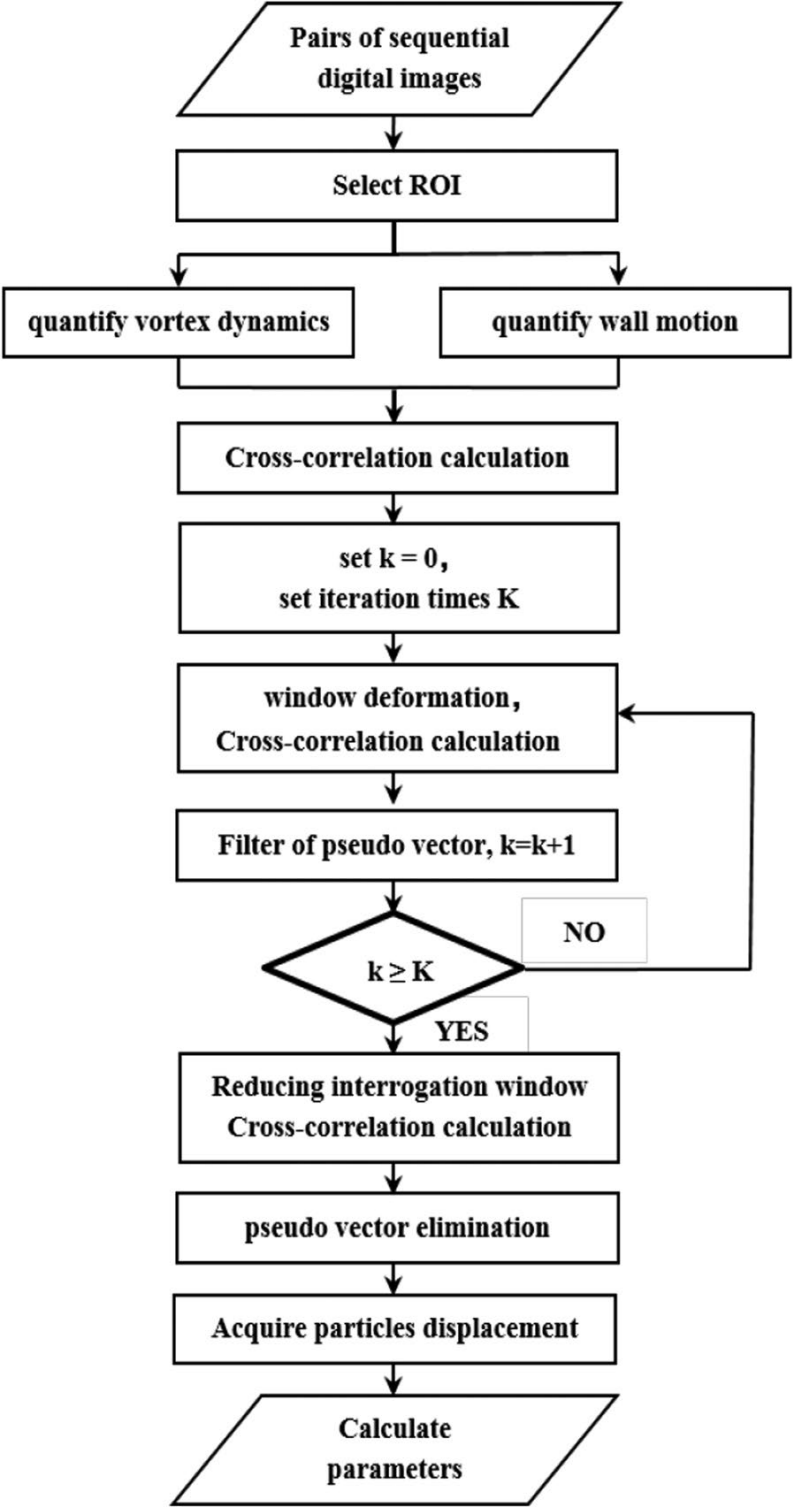
Flowchart of our ultrasonic biomechanics algorithm for left ventricular diagnostics

Figure 1 illustrates the schematic of our ultrasonic biomechanics algorithm. At the initial stage, the two-dimensional cross-correlation algorithm is used to compute the displacement of each interrogation window. Then, the proposed method integrates several iterative algorithms that are suitable candidates for calculating high-velocity gradient flows in the presence of velocity vector, the gradient of displacement, and the estimation of geometrical transformations of LV wall by strain rate, and the gradient of velocity. Finally, the size of interrogation window is reduced from squares of 52 × 52 pixels to 26 × 26 pixels, and the cross-correlation algorithm is applied with the reduced interrogation window to acquire higher spatial resolution. Similarly, spurious vector elimination algorithm is adopted to acquire exact evaluation of the displacements through the median filter.

### Parameter definition

Once the velocity field was acquired, the computed results were then rearranged in terms of LV fluid dynamics and wall motion parameters such as vorticity (which was a principal quantity to accordingly describe intraventricular blood flow patterns) and strain that gives a description of ventricular myocardial deformation. Generally, the parameters were measured by calculating the mean value in each interrogation window.

Mathematically as presented by [24], vorticity *ω*(*i*,*j*,*t*) is defined by the curl of velocity field

1 $$\omega (i,j,t) = \frac{{v_{y} (i + 1,j) - v_{y} (i - 1,j)}}{x(i + 1,j) - x(i - 1,j)} - \frac{{v_{x} (i,j + 1) - v_{x} (i,j - 1)}}{y(i,j + 1) - y(i,j - 1)} = \frac{{\partial v_{y} }}{\partial x} - \frac{{\partial v_{x} }}{\partial y},$$

where the units are as follows: *x* (m), *y* (m), *v*_*x*_ (m/s), and *v*_*y*_ (m/s).

Then, based on [3], the total vortex vorticity is computed as:

2 $$\Omega_{0} = \int\limits_{vortex} {\omega_{o} (x,y)dxdy} ,$$

The relative strength (*RS*) of pulsatile contribution with respect to the time-average flow is a measure of flow “vitality”, a global measure generated by [9]:

$$RS = \frac{1}{{\Omega_{0} }}\int\limits_{vortex} {\omega_{1} (x,y)dx,dy} .$$

where *ω*_0_(*x*,*y*) and *ω*_1_(*x*,*y*) represent the vorticity strength of the first and zeroth order Fourier harmonic, respectively. The first order is the main pulsatile contribution, while the zeroth order is the steady contribution.

In general terms, strain represents relative deformation and strain rate represents the rate of deformation. Therefore, they can be defined as:

4 $$S = \frac{{L - L_{0} }}{{L_{0} }} = \frac{\Delta L}{{L_{0} }},$$

where the length (*L*) is the instantaneous maximum width of the left ventricle, while *L*_*0*_ is the original length, and ∆*L* is the difference in length.

5 $$SR = \frac{s}{\Delta t} = \frac{{v_{2} - v_{1} }}{d},$$

And where *v*_2_ − *v*_1_means the change in instantaneous myocardial *v*_1_ and *v*_2_ at two points, distance d means change in velocity points at specific time.

### In vitro experimental model

**Fig. 2:**
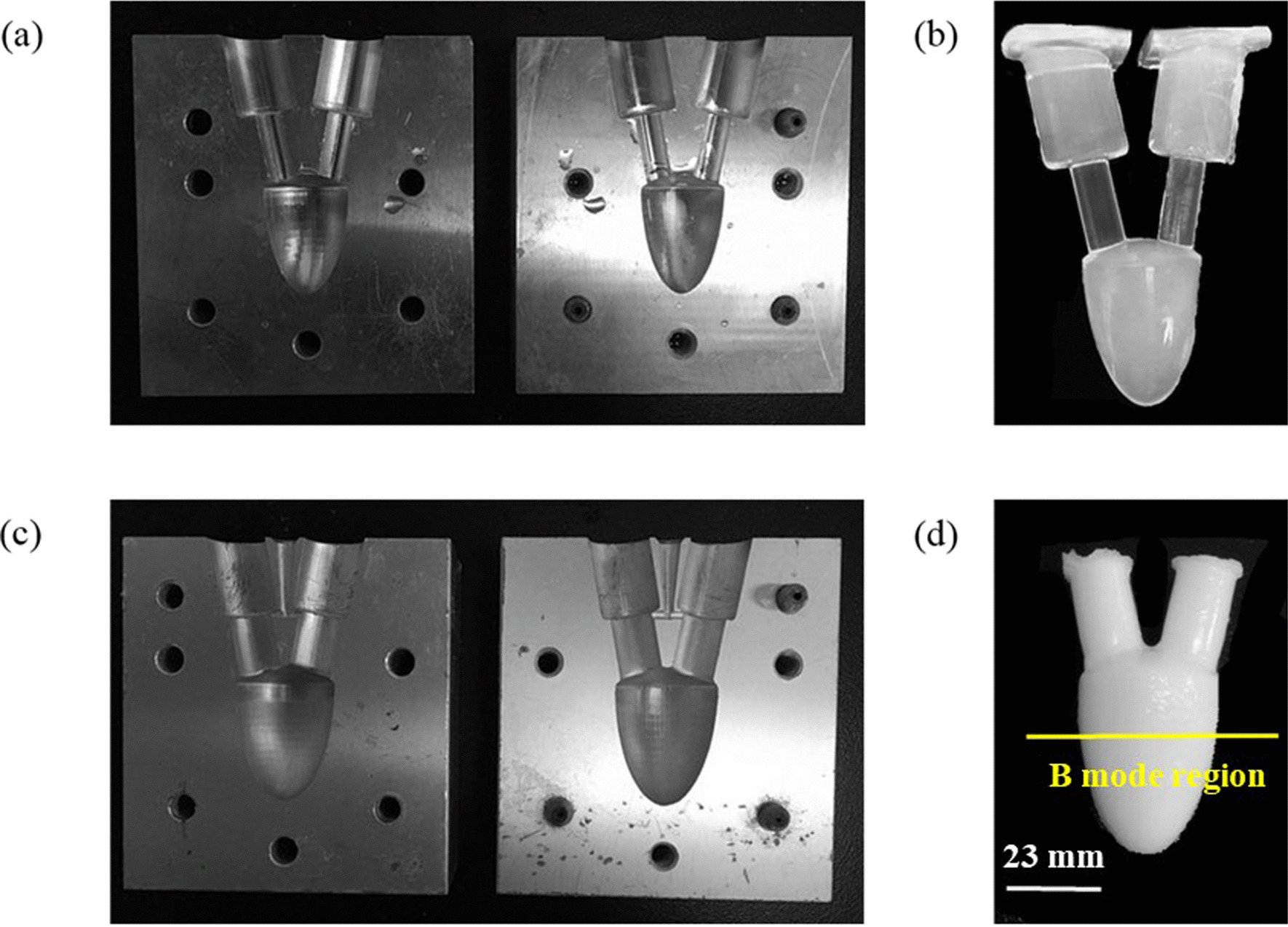
Photorealistic images of **a**, **c** metal molds with different sizes and **b**, **d** their respective left ventricular phantoms

**Fig. 3:**
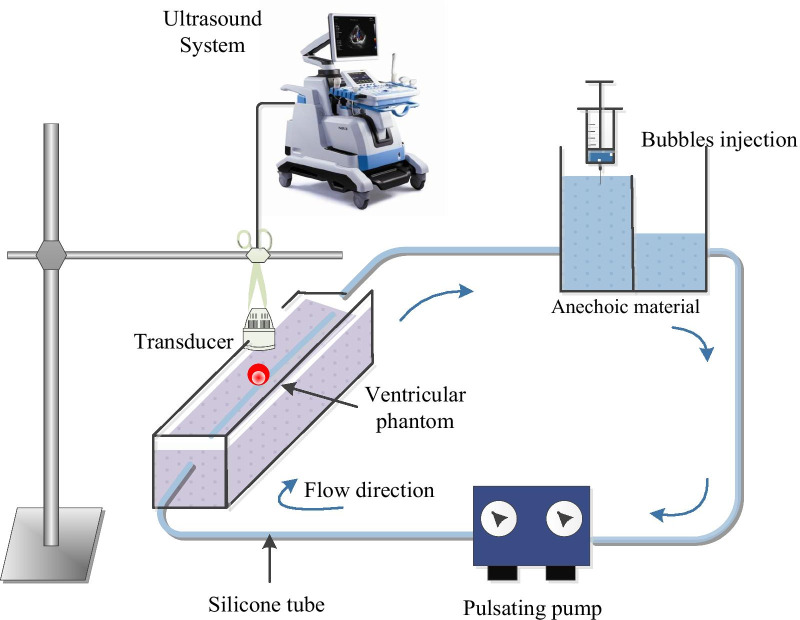
Experimental set-up used to measure the flow vortex and wall motion pattern of PVA

To accomplish this study, a LV pulsed flow simulator system was established. It comprised a LV phantom container with different stiffness, made from polyvinyl alcohol cryogen (PVA). PVA phantoms were made by two metal molds of different diameters, as shown in Fig. 2. In order to form a periodic, pulsatile flow of circulatory system, the pulsatile pump (Model 55-3305, Holliston, MA, USA) was set at a heart rate of 52 beats min^−1^, and the pump provided a waveform that simulates the realistic ventricular behavior. The waveform reproduced an output phase ratio of systole and diastole of 35/65. A 10 MHz linear array transducer (L14-5W/60) was connected to the Sonix RP ultrasound system (Ultrasonix Medical Corporation, Richmond, BC, Canada) laterally, scanning the left ventricular PVA phantom. Pairs of B-mode images, separated by a short time interval, were obtained with a frame rate of 97 Hz, and about 112 frames in one cycle. We captured images of the simulated blood flow by injecting ultrasound contrast microbubbles (UCAs) into the system and seeding them, which are supposed to faithfully follow the dynamics of fluid flow, into upstream chamber that are necessary for measure of displacement and related properties in fluids. To track the wall motion, scatters were mixed in the PVA when phantom was made. All the above mentioned presents the investigated working conditions for phantom experiments, as shown in Fig. 3.

### In vivo LV ultrasound image acquisition

This study was performed using a number of subjects including four patients with LV dysfunction (3 males, 1 females, averaged age 64.2 ± 5.5 years old, LV ejection fraction 56.0 ± 4.0%, all with coronary heart disease) and five healthy volunteers (3 males, 2 females, averaged age 40.0 ± 5.9 years old, LV ejection fraction 68.4 ± 2.1%). This study was approved by the Ethical Committee of the Third Affiliated Hospital of Sun Yat-sen University with waiver of informed consent. In this study, 1 MHz transducer was used to image all volunteers and patients at a frame rate of 30 to 60 Hz. Flow dynamics and myocardial contours were manually traced in different levels of view, and the echocardiography with contrast agent imaging was performed on volunteers to calculate LV blood flow dynamics in the apical 4-chamber views during one cardiac cycle. B-mode images were recorded from the left ventricular short-axis view (papillary muscle level) over three cardiac cycles to estimate LV myocardial motion. Note that the resolution in the direction that is perpendicular to the propagation direction of ultrasound beam tends to be low, so the direction of LV long axial ought to be parallel to the ultrasound beam for accurate strain measurements. All these echocardiography were stored in format of cine loop for succeeding offline proceeding and analysis. Images were processed using our proposed biomechanics algorithm as mentioned above.

### Statistical analysis

All statistical analyses were performed using the Statistical Package for Social Sciences statistical software package, version 17.0 (SPSS Inc., Chicago, IL, USA). A *p* value less than 0.05 was accepted as indicating statistical significance. Parametric data were expressed as mean ± standard. The analysis of covariance (ANOVA) was used to examine the difference of four parameters of vortex dynamics (DI, KEF, RS, and W) based on different elasticity of phantom. The *t* test of independent samples was used to compare the vorticity and wall shear stress between the normal and patient subjects.

## Results

### In vitro LV results

**Fig. 4:**
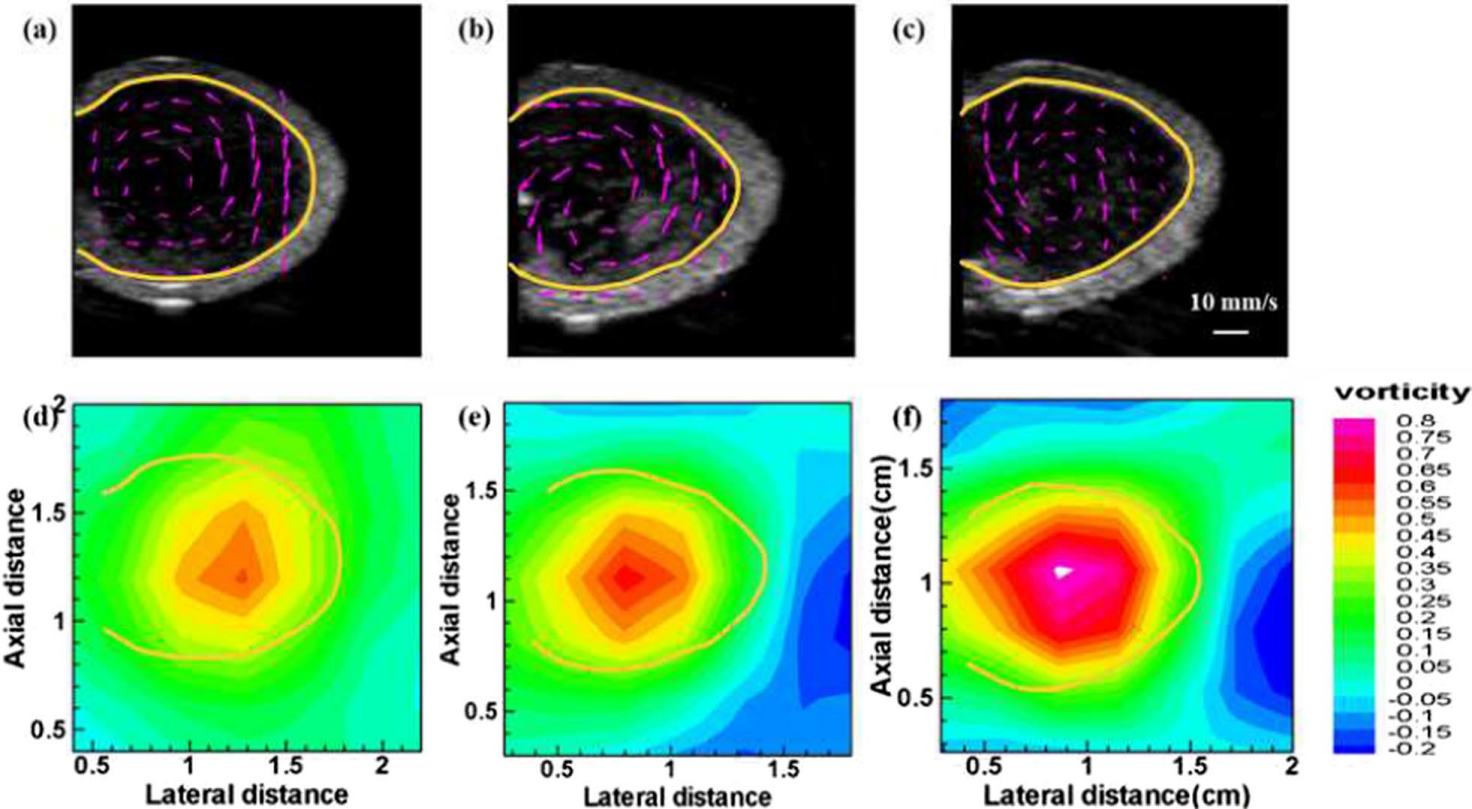
The vortex pattern of different elasticity modulus given by 169.79 kPa (**a**), 252.34 kPa (**b**), and 304.42 kPa (**c**); with their respective distribution of vortex of different elasticity modulus(**d**–**f**)

The velocity field of PVA phantom with different stiffness, which corresponds to the distinct elasticity modulus, was obtained by our biomechanics algorithm [25] as shown in Fig. 4 (top row). The purple arrows indicate the velocity vector, and the overall arrangement displays the diastolic vortex flow velocity pattern. The relevant spatial distributions of vorticity were computed at frame with vortex pattern as depicted in Fig. 4 (bottom row). There were significant differences between the vorticities of different phantoms, and the consequences exhibit a distinct increase in vorticity with increasing elastic modulus. Vorticity versus elasticity modulus shows a positive correlation.

**Fig. 5:**
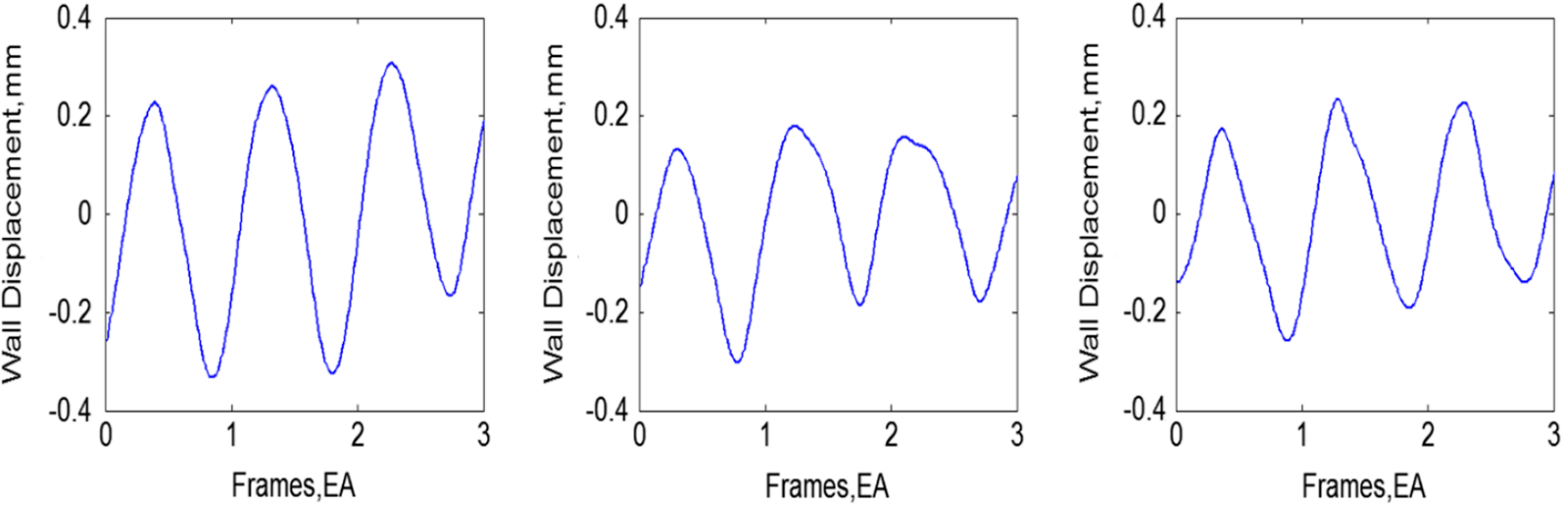
The measured wall displacement of LV phantom with different elasticity modulus given by 169.79 kPa, 252.34 kPa, and 304.42 kPa

For the evaluation of strain of wall motion, displacements were calculated along the axial direction that are parallel to the direction of propagation of ultrasound beam, as represented by our algorithm. The calculation results of the tracking wall segments in studies is shown in Fig. 5. Apparently, the peak of strain for wall motion decreased with the increase in elasticity modulus and periodically changed values. The statistical analysis for four parameters of vortex dynamic (DI, KEF, RS and W), at different phantom Elasticity (E) is shown in Table 1. These results depict the good viability and performance of the proposed algorithm in evaluation of LV function and pave a way to further studies in vivo.

**Table 1:** Statistical analysis for four parameters of vortex dynamics (DI, EF, RS, and W) based on different elasticity (E) of phantom

E (kPa)	DI	KEF	RS	W
169.79	0.701 ± 0.422	0.001 ± 0.000	0.412 ± 0.007	0.738 ± 0.030
252.34	0.754 ± 0.238	0.001 ± 0.000	0.424 ± 0.032	0.744 ± 0.013
304.42	0.818 ± 0.745	0.002 ± 0.000	0.635 ± 0.076	0.816 ± 0.079
F	3.935	45.500	20.724	2.325
*p*	0.081	< 0.001	0.002	0.179

Data are mean ± standard deviation

### In vivo LV results

**Fig. 6:**
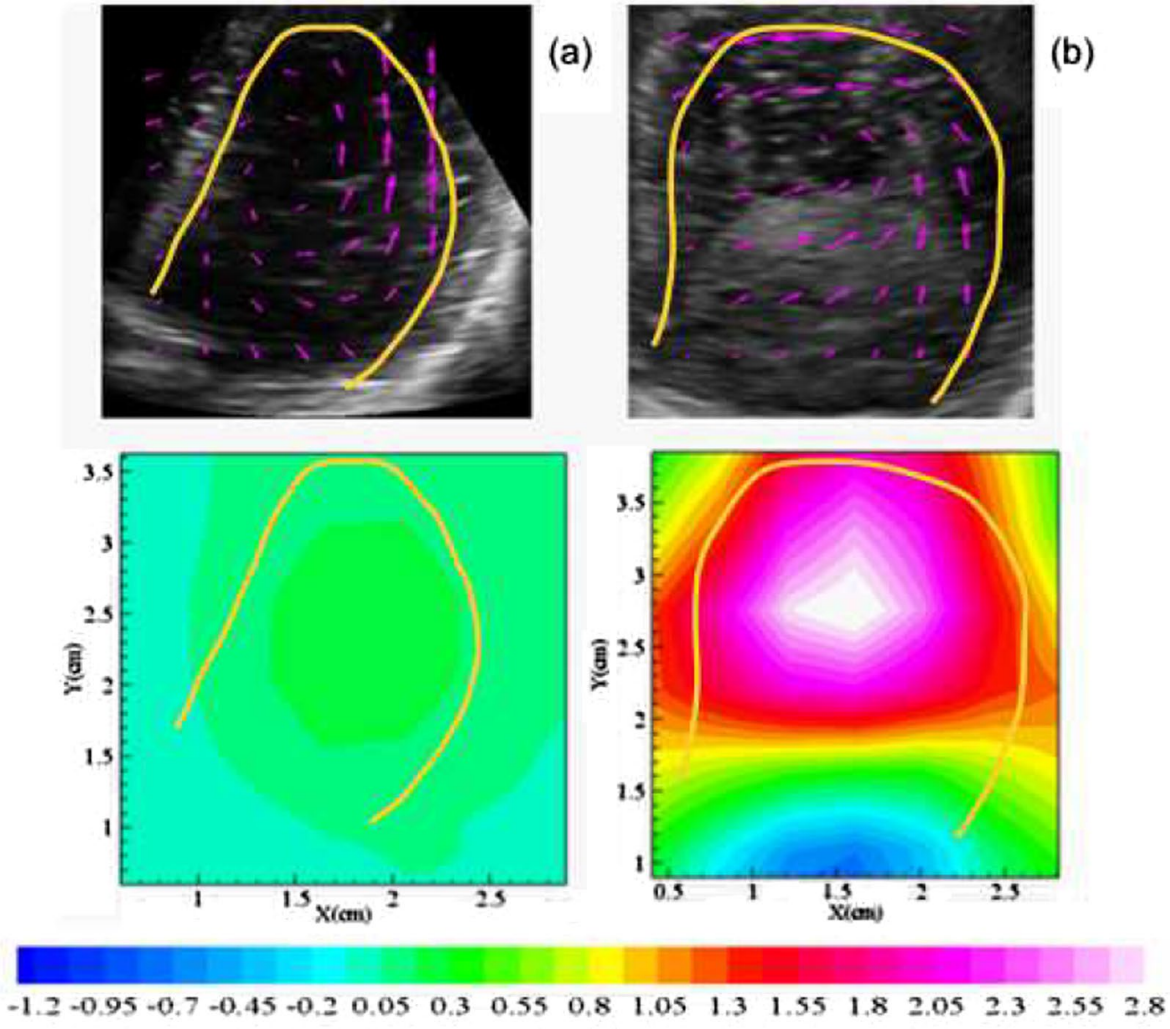
Vortex pattern of healthy volunteer (**a**) and patient with LV dysfunction (**b**) and vortices distribution (under)

The same ultrasonic biomechanics algorithm is implemented to process the images captured from human left ventricle. The average and maximum values of W and WSS of patients with LV dysfunction were larger than that of the control group, though the differences were not statistically significant (Table 2). Figure 6 reveals the flow pattern in human left ventricle, and a vortex structure. Moreover, it is observed that intraventricular flow patterns are distinctively discriminated in patients with LV dysfunction from normal subjects. We observed that the vortex pattern of a healthy volunteer was more pronounced in comparison to a patient with LV dysfunction, with values of 0.8 ms^−1^ versus 0.1 ms^−1^, which correlates to the strong versus weak elasticity of their left ventricle.

**Table 2:** Statistical analysis for W and WSS between normal and patients with LV dysfunction

	Average of W	Maximum of W	Average of WSS	Maximum of WSS
Normal (n = 5)	0.0870 ± 0.0145	0.3838 ± 0.1235	2.5337 ± 0.6863	6.1808 ± 1.6832
Patients (n = 4)	0.3217 ± 0.5215	0.9861 ± 1.7595	10.9111 ± 5.7678	26.1429 ± 16.0799
*t*	− 0.900	− 0.683	− 2.889	− 2.472
*p*	0.434	0.543	0.061	0.088

Data are mean ± standard deviation

**Fig. 7:**
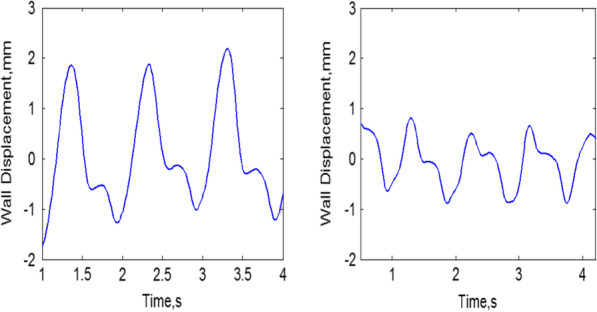
Measured wall displacement of healthy volunteer (left) with range from − 1.8 to 2.1 mm, and patient with LV dysfunction (right) with range from − 0.9 to 0.95 mm

To further optimize the detection of LV myocardial motion, it is recommended to capture the images when the ultrasound beam is parallel to the interventricular septum. As a result, the longitudinal strain equates to the axial component strain in the ultrasound coordinate system, as illustrated in Fig. 7. We note that the strain of a healthy elastic ventricle ranges from − 1.8 to 2.1 mm with an average of 1.2 mm, whereas that of a patient with LV dysfunction ranges from − 0.9 to 0.95 mm with an average of 0.9 mm, showing a 0.3 mm difference in the strain averages.

## Discussion

This study proposed a novel ultrasonic biomechanics method to simultaneously measure LV vortex and ventricular wall motion to assess LV function, which exhibits potential clinical value. For potential validation and suitability of real echocardiogram, the proposed algorithm was first tested in a phantom experimental set-up as a mean to access LV function. The measurement of these function parameters can contribute to characterizing LV related performance in normal subjects versus patients with LV dysfunction.

Previous studies focused merely on investigations to evaluate LV behavior from the aspect of either fluid dynamics or myocardial in left ventricle [26, 27]. In this study, we carried out ultrasonic imaging of the blood flow and elastic wall motion of left ventricular in both phantom and human study, and then combined the obtained vortex and wall motion of LV for dysfunction analysis. After collecting images, velocity vector or displacement vector for particle imaging velocimetry of blood flow and elastic wall imaging were estimated through cross-correlation algorithm, respectively [28–30]. Aided with sub-pixel method, median filtering of pseudo vector, linear interpolation and multiple iterative algorithms, the accuracy of the algorithm can be further improved.

In vitro study, the calculated velocity vector distribution could show the vorticity and wall shear force distribution at the moment of the vortex. The peak occurred at the central position of the vortex, and the peak showed a significant trend of increasing, which corresponded to the different elasticity modulus. The peak of strain for wall motion decreased with the increase in elasticity modulus and periodically changed values. Statistical analysis for parameters of vortex dynamics at various E of phantom depicted good viability of this algorithm.

In vivo study, the results confirmed that subjects with LV dysfunction had lower vorticity and S compared to the normal group. The peak of the vorticity and wall shear force occurred at the central position of the vortex in both groups, and the data in the patients with LV dysfunction showed larger values. The measured wall displacement of healthy volunteer was larger than that of patients with LV dysfunction. Also the W and WSS of patients with LV dysfunction were larger than that of the normal individuals, yet the difference was not statistically significant.

The potential for quantifying left ventricular function by eddy dynamics and wall motion was investigated in vitro. The current results show that eddy flow dynamics and wall motion characteristics are greatly affected by elasticity, and can be utilized as quantitative parameters in future analysis. It is verified that the two parameters are greatly affected by chamber elasticity, so as to quantify the difference of left ventricular eddy current and wall motion between the left ventricular dysfunction and control group. From the current results, we may speculate that the left ventricular dysfunction will be directly manifested in these two characteristic parameters, which tell noticeable discrepancies, and provide a reference tool for the diagnosis, prognosis and treatment of left ventricular dysfunction.

There were several limitations of this study: (1) Compared to the perpendicular-to-axial direction, this method is more accurate in axial direction to calculate wall motion, parallel to the direction of ultrasonic beam. The reason might be the low resolution of the ultrasonic probe in this direction. (2) Given the small number of vivo cases, our findings must be regarded as preliminary. Future research with larger sample sizes are needed to verify these results.

## Conclusion

This work proposes a novel algorithm to examine LV function from echocardiogram that is adapted to both vortex dynamics and wall motion. In vitro phantom study demonstrated the potential of our new method to detect abnormal vortex and wall motion. The current data in vivo have shown the difference in parameters of both vortex dynamics and wall motion between normal subjects and patients with LV dysfunction. The deviation in vorticity and strain is correlated with LV’s mechanical performance and this method may have potential clinical value in evaluation of LV dysfunction.

## Data Availability

The datasets used and/or analysed during the current study available from the corresponding author on reasonable request.

## Abbreviations

LV: Left ventricle
PVA: Polyvinyl alcohol cryogen
WSS: Wall shear stress
DTI: Tissue Doppler imaging
ROI: Region of interest
RS: Relative strength
E: Elasticity
DI: Energy dissipation index
KEF: Kinetic energy fluctuations
RS: Relative pulsatile vorticity strength
W: Vorticity
S: Strain

## References

[1] Solomon SD, Zelenkofske S, Mcmurray JJV (2005). Sudden death in patients with myocardial infarction and left ventricular dysfunction, heart failure, or both. N Engl J Med.

[2] Petersen JW, Nazir TF, Lee L, Garvan CS, Karimi A (2013). Speckle tracking echocardiography-determined measures of global and regional left ventricular function correlate with functional capacity in patients with and without preserved ejection fraction. Cardiovasc Ultrasound.

[3] Hong G-R, Pedrizzetti G, Tonti G (2008). Characterization and quantification of vortex flow in the human left ventricle by contrast echocardiography using vector particle image velocimetry. JACC Cardiovasc Imaging.

[4] Kim WY, Walker PG, Pedersen EM (1995). Left ventricular blood flow patterns in normal subjects: a quantitative analysis by three-dimensional magnetic resonance velocity mapping. J Am Coll Cardiol.

[5] Gharib M, Rambod E, Kheradvar A, Sahn DJ, Dabiri JO (2006). Optimal vortex formation as an index of cardiac health. Proc Natl Acad Sci USA.

[6] Firstenberg MS, Vandervoort PM, Greenberg NL (2000). Noninvasive estimation of transmitral pressure drop across the normal mitral valve in humans: importance of convective and inertial forces during left ventricular filling. J Am Coll Cardiol.

[7] Kilner PJ, Yang GZ, Wilkes AJ, Mohiaddin RH, Firmin DN, Yacoub MH (2000). Asymmetric redirection of flow through the heart. Nature.

[8] Cenedese A, Prete ZD, Miozzi M, Querzoli G (2005). A laboratory investigation of the flow in the left ventricle of a human heart with prosthetic, tilting-disk valves. Exp Fluids.

[9] Agati L, Cimino S, Tonti G, et al. Quantitative analysis of intraventricular blood flow dynamics by echocardiographic particle image velocimetry in patients with acute myocardial infarction at different stages of left ventricular dysfunction. Eur Heart J Cardiovasc Imaging. 2014; 15(11):1203–12. 10.1093/ehjci/jeu106 24906998

[10] Niu L, Qian M, Song R, Meng L, Liu X, Zheng H (2012). A 2D non-invasive ultrasonic method for simultaneous measurement of arterial strain and flow pattern. Clin Physiol Funct Imaging.

[11] Kheradvar A, Pedrizzetti G (2000). Vortex formation in the cardiovascular system. J Differ Equ.

[12] Buckberg GD, Hoffman JI, Coghlan HC, Nanda NC. Ventricular structure-function relations in health and disease: Part II. Clinical considerations. Eur J Cardio-Thoracic Surg Off J Eur Assoc Cardio-thoracic Surg. 2014; 47(5). 10.1093/ejcts/ezu279 25082144

[13] Urheim S, Edvardsen T, Torp H, Angelsen B, Smiseth OA. Myocardial strain by Doppler echocardiography. Validation of a new method to quantify regional myocardial function. Circulation. 2000; 102(10):1158–64. 10.1161/01.cir.102.10.1158 10973846

[14] Marwick TH (2006). Measurement of strain and strain rate by echocardiography: ready for prime time?. J Am Coll Cardiol.

[15] Leitman M, Lysyansky P, Sidenko S (2004). Two-dimensional strain-a novel software for real-time quantitative echocardiographic assessment of myocardial function. J Am Soc Echocardiogr.

[16] D'Hooge J, Heimdal A, Jamal F (2000). Regional strain and strain rate measurements by cardiac ultrasound: principles, implementation and limitations. Eur J Echocardiogr.

[17] Sutherland GR, Salvo GD, Claus P, D'Hooge J, Bijnens B (2004). Strain and strain rate imaging: a new clinical approach to quantifying regional myocardial function. J Am Soc Echocardiogr.

[18] Voigt JU, Arnold MF, Karlsson M (2000). Assessment of regional longitudinal myocardial strain rate derived from doppler myocardial imaging indexes in normal and infarcted myocardium. J Am Soc Echocardiogr.

[19] Miyatake K, Yamagishi M, Tanaka N (1995). New method for evaluating left ventricular wall motion by color-coded tissue Doppler imaging: in vitro and in vivo studies. J Am Coll Cardiol.

[20] Takigiku K, Takeuchi M, Izumi C (2012). Normal range of left ventricular 2-dimensional strain: Japanese Ultrasound Speckle Tracking of the Left Ventricle (JUSTICE) study. Circ J.

[21] Delgado V, Ypenburg C, van Bommel RJ (2008). Assessment of left ventricular dyssynchrony by speckle tracking strain imaging comparison between longitudinal, circumferential, and radial strain in cardiac resynchronization therapy. J Am Coll Cardiol.

[22] Curiale AH, Sánchez-Ferrero GV, Aja-Fernández S (2016). Influence of ultrasound speckle tracking strategies for motion and strain estimation. Med Image Anal.

[23] Niu L, Qian M, Wan K (2010). Ultrasonic particle image velocimetry for improved flow gradient imaging: algorithms, methodology and validation. Phys Med Biol.

[24] Abe H, Caracciolo G, Kheradvar A (2013). Contrast echocardiography for assessing left ventricular vortex strength in heart failure: a prospective cohort study. Eur Heart J Cardiovasc Imaging.

[25] Niu L, Qian M, Song R (2012). A texture matching method considering geometric transformations in noninvasive ultrasonic measurement of arterial elasticity. Ultrasound Med Biol.

[26] Kheradvar A, Houle H, Pedrizzetti G (2010). Echocardiographic particle image velocimetry: a novel technique for quantification of left ventricular blood vorticity pattern. J Am Soc Echocardiogr.

[27] Chetboul V, Serres F, Gouni V, Tissier R, Pouchelon J-L (2007). Radial strain and strain rate by two-dimensional speckle tracking echocardiography and the tissue velocity based technique in the dog. J Vet Cardiol.

[28] Willert CE, Gharib M (1991). Digital particle image velocimetry. Exp Fluids.

[29] Voorneveld J, Muralidharan A, Hope T (2017). High frame rate ultrasound particle image velocimetry for estimating high velocity flow patterns in the left ventricle. IEEE Trans Ultrason Ferroelectr Freq Control.

[30] Voorneveld J,Keijzer LBH,Strachinaru M,et al. High-frame-rate echo-particle image velocimetry can measure the high-velocity diastolic flow patterns.Circulation Cardiovasc Imaging. 2019; 12(4): e008856. 10.1161/CIRCIMAGING.119.008856 30939921

